# Fermentation for Revalorisation of Fruit and Vegetable By-Products: A Sustainable Approach Towards Minimising Food Loss and Waste

**DOI:** 10.3390/foods13223680

**Published:** 2024-11-19

**Authors:** José Ángel Salas-Millán, Encarna Aguayo

**Affiliations:** 1 Postharvest and Refrigeration Group, Universidad Politécnica de Cartagena (UPCT), Paseo Alfonso XIII, 48, 30203 Cartagena, Spain; encarna.aguayo@upct.es; 2 Food Quality and Health Group, Institute of Plant Biotechnology (IBV-UPCT), Campus Muralla Del Mar, 30202 Cartagena, Spain

**Keywords:** lactic acid bacteria, kombucha, kefir, phytochemical, antioxidant, functional, fortified foods, probiotic, pickled, fruit-vine, vinegar

## Abstract

In a world increasingly focused on sustainability and integrated resource use, the revalorisation of horticultural by-products is emerging as a key strategy to minimise food loss and waste while maximising value within the food supply chain. Fermentation, one of the earliest and most versatile food processing techniques, utilises microorganisms or enzymes to induce desirable biochemical transformations that enhance the nutritional value, digestibility, safety, and sensory properties of food products. This process has been identified as a promising method for producing novel, high-value food products from discarded or non-aesthetic fruits and vegetables that fail to meet commercial standards due to aesthetic factors such as size or appearance. Besides waste reduction, fermentation enables the production of functional beverages and foods enriched with probiotics, antioxidants, and other bioactive compounds, depending on the specific horticultural matrix and the types of microorganisms employed. This review explores the current bioprocesses used or under investigation, such as alcoholic, lactic, and acetic acid fermentation, for the revalorisation of fruit and vegetable by-products, with particular emphasis on how fermentation can transform these by-products into valuable foods and ingredients for human consumption, contributing to a more sustainable and circular food system.

## 1. Introduction

The revalorisation of horticultural by-products is seen as a key strategy to minimise loss and waste and maximise the value of the food supply chain in a world that is increasingly aware of sustainability and integrated resource use. Revalorisation strategies cover all the processes involved in converting by-products or residues into new high-value products, while the food supply chain includes agricultural production, post-harvest operations, processing, storage, transport, wholesale/retail, and consumption. Food loss refers to the decrease in the quantity or quality of food along the supply chain, from production up to, but not including retail level. These losses mainly occur during the production, post-harvest, and processing stages. Food waste is the discarding of food that is fit for consumption at retail or consumer level, and is often due to decisions related to storage, handling, or consumer behaviour [1]. Addressing food losses is essential to reducing hunger, increasing agricultural yields, and improving food security. The causes of food loss vary globally and are influenced by factors such as production methods, choices, infrastructure, market dynamics, and consumer practices [2]. However, the main causes of food waste are related to the shelf-life, the lack of cosmetic standards such as size, colour, and shape, and fluctuations in demand [3]. Food loss and waste do not only have a direct economic impact on farmers and consumers, but also negatively impact the environment. Resources such as land, water, and energy used in production are wasted, thus leading to unnecessary CO_2_ emissions.

**Figure 1:**
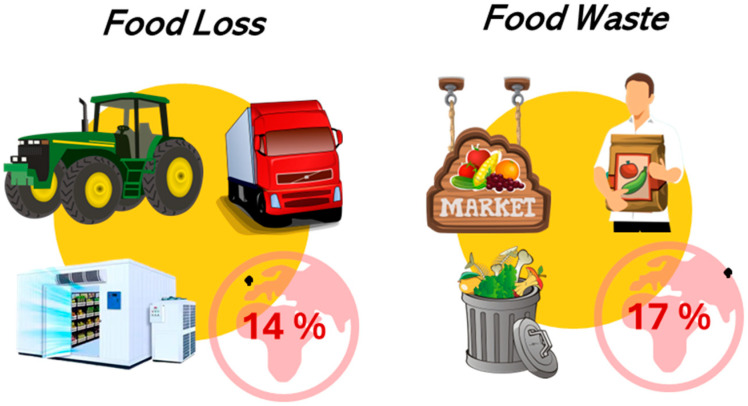
Food loss and food waste designation of food residues and their impact on the food supply chain [1].

Distinguishing between food loss and food waste is crucial since interventions that target consumer behaviour (food demand) differ from those that encourage suppliers to minimise food loss (food supply). Between the producer and the consumer an estimated 13.8% of the food produced is lost, and about 17% of the food available at consumer level is wasted globally (11% in households, 5% in food services, and 2% in retail; Figure 1) [4]. Different factors influence the extent of food loss and waste at each level of the food supply chain. At the primary production level, agricultural practices have a significant impact on outcomes. For example, sharp declines in market prices or strict quality standards can lead to substantial quantities of fruits and vegetables being left unharvested. Additionally, direct causes such as suboptimal agronomic practices, choice of crop varieties, machinery failures, and inadequate labour practices can contribute to increased food loss during harvesting. Deficiencies in storage and transportation infrastructure, inadequate temperature and humidity control, prolonged storage periods, and logistical mismanagement also contribute to food loss. During the processing and packaging stages, inadequate processing capacity, technical malfunctions and the pursuit of specific aesthetic standards can result in increased losses. For instance, excessive trimming to meet cosmetic standards can significantly increase the volume of discarded produce. In the context of the wholesale and retail sectors, both indirect factors such as fluctuations in the demand for perishable products, and direct causes such as inappropriate product displays, removal of “imperfect” looking goods, and overstocking are of critical importance. With respect to households and food services, the confusion between expiry date and best before date labels, along with poor storage practices and oversized portions, represent direct causes of fruit and vegetable waste [1].

Several international and national policies have been implemented with the objective of reducing food waste. Initiatives such as the “Transforming Our World: the 2030 Agenda for Sustainable Development”, the “Champions 12.3 Initiative”, and the “Food Loss and Waste Initiative” (2015) underscore the need for a collaborative approach involving governments, businesses, NGOs, and society to address food loss and waste at various production levels. Furthermore, such initiatives provide invaluable insight into the current state of research and innovations within this field [5]. At national level, the United Kingdom has launched the Courtauld Commitment, which aims to make food and drink production more sustainable [6]. In France, significant policy measures enacted in 2015 prohibit supermarkets from discarding unsold food, mandating it be donated to charities and food banks instead [7]. Similarly, Spanish re-search initiatives are increasingly focusing on funding research projects related to the circular economy and strategies for revalorisation to mitigate food waste [8].

**Figure 2:**
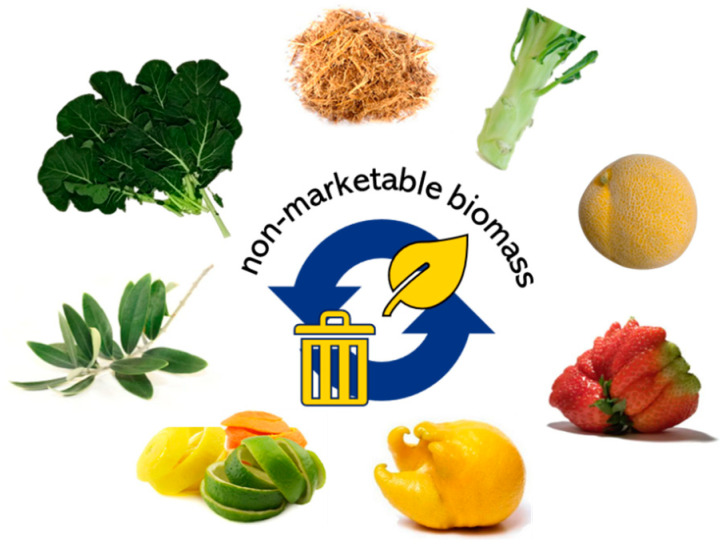
Non-marketable by-products from crops and industrial food production.

In addition to food loss and waste, food production also generates various edible or non-edible parts that are not marketable, such as leaves, stems, peels, bark, and pulp from fruits and vegetables (Figure 2). These non-marketable products, often considered as bio-residues, are frequently discarded before they can be revalorised. Therefore, the transformation of these non-marketable bio-residues, now considered by-products, could realise their potential as a health-promoting and functional source, enabling their incorporation to fortify foods or in the development of novel foods [9]. The biomass discarded from food loss, waste and non-marketable parts has significant nutritional potential value, and includes proteins, carbohydrates, fats, and minerals, as well as bioactive compounds beneficial to health, such as polyphenols, fibres, glucosinolates, vitamins, etc. These compounds have been linked reduced incidence of degenerative diseases, including certain types of cancers, ageing, cardiovascular diseases, and diabetes, amongst others. Bioactive compounds are phytochemicals found in fruits and vegetables that have physiological properties beyond nutritional considerations [10].

Fermentation has a long history as a food processing technique for improving the nutritional value, digestibility, safety, and taste, by using microorganisms or enzymes to induce desirable biochemical changes. The global community contains very different dietary habits that have been shaped by unique cultures, religious beliefs, and the availability of food resources [11]. However, although fermented foods are also steeped in their own culture and history, the rise of more industrially processed food products has led to a reduction in their production and diversity, particularly in West-ern countries. However, the growing interest in health-promoting foods, artisan-like processes, and their promotion in national dietary recommendations has contributed to the increasing popularity of fermented foodstuffs in Western diets in recent years [12]. Fermentation processes have been improved by advances in microbiology and biotechnology, as well as by the development of starter cultures [13]. The fermentation capabilities of different microorganisms vary considerably. When these are employed in conjunction with diverse food matrices, the resulting products exhibit a range of valuable characteristics, including a significant reduction in sugar content, modified texture, aroma profiles, and flavours, improved nutritional value, potential for ex-tended shelf life [14], and probiotic effects that enhance gut health [11]. In terms of the food matrix, fermented foods could be classified as dairy products, fermented cereals, vegetables, fruits, legumes, or meat- and fish-based products. Although fermentation is a natural process that occurs through microbial action, different chemical extraction methods have also been extensively studied for their efficiency in recovering health-promoting phytochemicals from food waste. For example, ultrasonic extraction, supercritical fluid extraction, pulsed electric field technology, high hydrostatic pressure, and microwave extraction, among others, offer advantages in terms of their speed and efficiency [15] but fail to provide the specific health benefits associated with live probiotic cultures found in certain fermented products. In this context, the revalorisation of fruit and vegetable by-products through fermentation is a sustainable approach towards minimising food loss and waste. As fermentation has been postulated as a promising way to obtain novel food products the present review outlines the different bioprocesses currently in use or under investigation for the revalorisation of fruit and vegetable by-products. Particular focus has been placed on the conversion of these by-products into new valuable foodstuffs and food ingredients.

## 2. Literature Review

The aim of this review is to provide an overview of updated evidence on the topic by compiling and evaluating the information from the Scopus and Web of Science bibliographic databases of scientific articles from the last 25 years (September 2024). Thus, the search was scoped considering the following key concepts: waste, residue, revalorisation, by-product, bioprocess, fermentation, food, and beverage. Only papers included in the Journal Citation Reports written in English, and which included experimental design and data treatment, were considered.

## 3. Microbial Diversity in the Transformation of Agricultural By-Products to New Foodstuffs

Traditionally, fermented foods have been produced by spontaneous fermentation (without a starter culture) or by the back-slopping method, in which a portion of the previous fermentation is added to the fresh substrate. However, in the last century, starter culture has been available and used in industrial fermentation to promote the fermentation in a scalable, reliable, consistent, and safe process [11]. The genera of fermented microorganisms used in fermented foods include yeasts such as *Saccharomyces*, *Zygosaccharomyces* and *Candida*; lactic acid bacteria (LAB) such as *Streptococcus*, *Lactobacillus*, *Lactiplantibacillus*, *Lactococcus*, *Leuconostoc*, *Staphylococcus*, *Pediococcus*, *Oenococcus*, and *Bacillus*; acetic acid bacteria such as *Acetobacter*, propionic acid bacteria such as *Propionibacterium*, and butyric acid bacteria such as *Clostridium* [12,14].

Some mould genera associated with fermented foods are used as starters in the food matrix preparation, such as *Rhizopus* spp. and *Aspergillus* spp. These moulds possess enzymatic activity such as amylolytic, lipolytic, and proteolytic functions, which are essential for nutrient assimilation by other microorganisms [16]. The most common genera and species in the fermented foods process are detailed in Table 1. Although LAB are generally preferred for the fermentation of dairy products, meat, vegetables, and legumes, the fermentation of fruits is primarily carried out by yeasts such as *Saccharomyces* spp., *Debaromyomyces* spp., amongst others. This is particularly relevant due to their superior ability to transform volatile compounds that enhance aroma, as we shall explain below.

In general, *Saccharomyces cerevisiae* and LAB are the currently relevant microorganisms in the industrial production of fermented foods and alcoholic beverages. *Saccharomyces cerevisiae* is widely used as a model organism in physiology, genetics, and molecular biology models and was the first eukaryotic organism to have its genome completely sequenced [17]. It is also the genus of yeast that has been used for centuries in baking, brewing, distilling, and in winemaking throughout human history. It is also found in dairy products such as yoghurt and cheese and in fermented vegetables [18]. Historically, bakery, brewing and winemaking products were produced by spontaneous fermentation by yeasts derived from the natural microflora of the substrate and environment (e.g., from the grape skins and the winery in the case of wine, and from cereals in the case of bread and beer). Although yeasts vary greatly, *S. cerevisiae* is the dominant species in these spontaneous fermentations [19]. Nevertheless, sometimes undesirable wild yeast strains can affect the organoleptic acceptance of wines and beers and may also vary the quality and homogeneity between production batches. Therefore, the use of commercial yeast strains became standard practice in industrial winemaking and brewing due to their practicality, better control of fermentation, and the favourable final product results [18,20]. During alcoholic fermentation, *S. cerevisiae* digests hexose and disaccharide sugars to ethanol and CO_2_ in an anaerobic environment, as well as other minor volatile metabolites that constitute the characteristic aroma profile and enhance the sensory properties in alcoholic beverages [18]. Fermentation significantly enhances the sensory profiles by modifying their volatile com-pound composition. In fermented beverages such as wine, the main volatile compounds include carbonyls, acids, terpenes, norisoprenoids, higher alcohols, acetate, and ethyl esters [21,22]. However, during alcoholic fermentation other undesirable volatile compounds are produced, including sulphur compounds that contribute to off-flavours. These compounds include carbon sulfide, ethanethiol, methanethiol, and hydrogen sulfide [23]. The Ehrlich pathway is responsible for the transformation of the aroma profile during alcoholic fermentation by yeast [24]. During the metabolism of *S. cerevisiae*, amino acids are transformed into new volatile compounds through trans-amination, decarboxylation and reduction to higher alcohols [3]. Finally, an esterification with acetyl-CoA promotes the generation of different types of acetate ester, de-pending on its structure from the precursor amino acids. Fatty acid ethyl esters (FAEEs) are also among the most common volatile compounds present following yeast metabolism in alcoholic fermentation. They are derived from fatty acids or fusel acids (from amino acid metabolism) esterified with ethanol [25]. Although aroma plays an important role in the quality of alcoholic beverages and other fermented foods, organoleptic characteristics also consider parameters such as physical (e.g., pH, density, col-our), chemical (e.g., ethanol, acidity, residual sugars), microbiological (e.g., presence of yeast, bacteria or contaminating microorganisms), and additional sensory parameters (e.g., intensity, authenticity), amongst others [26]. Furthermore, fermentation markedly improves the texture and nutritional value of a range of food products, resulting in enhancements to sensory attributes and potential health benefits. During fermentation, the microbial metabolism breaks down complex carbohydrates and proteins which can alter the texture of food products. This often results in a softer, more palatable consistency that consumers find appealing. For instance, fermentation can increase the digestibility of tough plant fibres, making the resulting products more enjoyable to eat and easier to digest, which complements the enhancements in the aroma and flavour profiles [27]. Thus, the total quality of fermented foods is influenced by several variables, suggesting the need for further study and evaluation in their development.

**Table 1:** Most common microbial genera and species in food fermentation: processes and applications across diverse food matrices.

Type of Fermentation	Microorganism Involved	Main Process	Food Matrix
Alcoholic	Yeast: *Saccharomyces* spp., *Debaryomyces* spp., *Candida* spp.	Transformation of sugars into alcohol and carbon dioxide	Wine, beer, cider, sourdough, bread, coffee, meat
Lactic acid	Bacteria: *Streptococcus* spp., *Lactobacillus* spp.	Conversion of lactose and other sugars into lactic acid	Dairy products, meats, vegetables, legumes
Propionic acid	Bacteria: *Propionebacterium* spp., *Acidipropionibacterium* spp.	Conversion of glucose into propionic acid, acetate, and carbon dioxide	Dairy products
Acetic acid	Bacteria: *Acetobacter* spp., *Gluconobacter* spp., *Bacillus subtilis* var. *Natto*,	Oxidisation of ethanol to produce acetic acid	Vinegar, nattō
Enzymatic activity	Fungi: *Aspergillus* spp., *Rhizopus* spp.	Breakdown of complex carbohydrates and proteins into simpler compounds	Soy sauces, soybean, rice wines, vinegars

Information based on [11,14,28,29,30].

The LAB group belongs to the order *Lactobacillales*, and are Gram-positive, cata-lase-negative, non-spore-forming bacteria that produce lactic acid. Genera in the LAB group include *Aerococcus*, *Carnobacterium*, *Enterococcus*, *Lactobacillus*, *Lactococcus*, *Leuconostoc*, *Oenococcus*, *Pediococcus*, *Streptococcus*, and *Weisella*, as listed in the List of Prokaryotic Names with Standing in Nomenclature (LPNS) [31] According to the new phylogeny based on whole-genome sequences [32], the families *Lactobacillaceae* and *Leuconostocaceae* have been merged into a single family, *Lactobacillaceae*, resulting in the reclassification of the genus Lactobacillus into 25 genera, including *Lactobacillus*, *Acetilactobacillus*, *Agrilactobacillus*, *Amylolactobacillus*, *Apilactobacillus*, *Bombilactobacillus*, *Companilactobacillus*, *Dellaglioa*, *Fructilactobacillus*, *Furfurilactobacillus*, *Holzapfelia*, *Lacticaseibacillus*, *Latilactobacillus*, *Lactiplantibacillus*, *Lapidilactobacillus*, *Lentilactobacillus*, *Levilactobacillus*, *Ligilactobacillus*, *Limosilactobacillus*, *Liquorilactobacillus*, *Loigolactobacilus*, *Paralactobacillus*, *Paucilactobacillus*, *Schleiferilactobacillus*, and *Secundilactobacillus* [32,33]. The European Food Safety Authority (EFSA) has assigned Qualified Presumption of Safety (QPS) status to many LAB species. This QPS status provides a harmonised, generic pre-assessment to support the safety risk assessment of biological agents intentionally introduced into the food and feed chain, as well as to facilitate applications for market authorisation [34].

Depending on their tolerance in aerobic or anaerobic conditions and its ability and flexibility to use oxygen, LAB could be classified as aerobic strict, which depend on oxygen for growth; anaerobic strict, which only tolerate free oxygen environments, and facultatively anaerobic, which can grow in both oxygen and oxygen-free environments. Lactic acid bacteria can grow in a temperature range of 5 to 45 °C and a pH range of 3.5 to 10 [35], where carbohydrates (hexoses and pentoses) are metabolised in three catabolic pathways (homofermentative, heterofermentative and facultative heterofermentative) to produce organic acids. The main known organic acids are lactic and acetic acids [36]. In addition, some LAB species can also produce other organic acids, for instance propionate, using glycerol as a carbon source to produce ATP, such as Enterococcus faecalis that was isolated from some fermented foods [37,38]. Some bacteria of the genus Propionibacterium also metabolise the carbon substrate and produce propionic acid as a final product during their fermentation process. *P. freudenreichii* is the most common species used in Swiss-type cheeses, but this genus of bacte-ria does not belong to LAB [39]. Other LAB, such as *Lactobacillus helveticus* (ATCC 15807), can produce acetate and succinate organic acids, depending on the external pH [40]. The production of these organic acids lowers the pH of the matrix during the fermentation, and this decrease can inhibit other bacterial competitors, protect from degradation, and extend the shelf life of food [35]. This is known as biopreservation, defined as a controlled bioprocess —usually involving LAB— and/or their antimicrobial secondary metabolites and bacteriocins to enhance the safety of food processing and extend the shelf life of finished products [41]. Several studies have evaluated the inhibitory effects of LAB against foodborne pathogens such as Salmonella spp., Listeria monocytogenes, and *Escherichia coli* [42]. The main mechanisms for biopreservation in fermented foods are the production of organic acids by LAB, and their ability to lower the pH. The acidic environment created by those acids leads to deformation and damage to the metabolism, enzymatic activities, proteins, and DNA structure of foodborne pathogens [43]. In addition, certain LAB strains are recognised for their ability to produce additional antimicrobial substances, including low molecular weight metabolites such as reuterin, reutericyclin, diacetyl, and fatty acids. They also produce hydrogen peroxide, antifungal compounds propionate, phenyl-lactate, hydroxyphenyl-lactate and 3-hydroxy fatty acids, as well as bacteriocins and bacteriocin-like molecules [44].

The highly available consortia of microorganisms present numerous opportunities for the creation of new and probiotic foods, particularly from fruit and vegetable by-products. It is important to highlight that the consumption of probiotics has been associated with many health benefits, including reduced cholesterol levels, modulation of the immune system, improved mineral absorption, decreased constipation, potential anticancer effects, antihypertensive effects, inhibition of pathogens, and modulation of the gut microbiota. Therefore, the potential for biotransformation of different matrices is huge, and several research studies have already provided valuable know-how to approach these processes and their associated health benefits [45].

## 4. Revalorisation of Fruit and Vegetable By-Products as Sources for the Fermentation Process

Fruit and vegetable by-products come mainly from two industrial sectors: the fresh produce trade and the processing industry. In both cases, a significant amount of fruit and vegetables and their parts are discarded, whether it be for aesthetic, economic, or processing reasons. In the fresh produce supply chain, a large volume of fruit is not marketed because it fails to meet the aesthetic standards demanded by consumers and supermarkets. These standards, which include size, shape, and colour criteria, generate significant waste as fruit that does not meet these requirements, despite being fit for consumption, is excluded from the fresh market [46]. It is estimated that be-tween 20% and 40% of produced fruit and vegetables are lost before reaching the final consumer due to such sorting criteria [47]. On the other hand, ready-to-eat, minimally processed or fresh-cut fruits and vegetables also generate significant amounts of by-products. During processing, parts such as peel, seeds, cores, and stalks are re-moved, whilst damaged fruit is discarded. In general, this can amount to 10 to 40% of the original whole product [48]. In addition, industries such as canned fruit and vegetable products, juice, jam, and puree production, along with alcoholic beverages such as wine and cider, also generate residues in the form of pulp, peels, and seeds, which can account for up to 30% of the total weight of processed fruits and vegetables [49]. These by-products, historically considered as waste, have gained increased interest due to their content in bioactive compounds such as antioxidants, amino acids, dietary fibres and other components with potential added value [50]. They can be used as ingredients in the production of functional products, nutraceuticals or as biomass to produce biofuels and other industrial products [51]. The present review focuses on the re-valorisation of by-products as a source during fermentation processes to obtain new products for human consumption.

### 4.1. Fruit By-Products

Fruit peels represent a significant source of by-products in the food industry, offering potential for revalorisation due to their content in biologically active molecules, including carotenoids, polyphenols, vitamins, fibres, and other compounds. This makes them a valuable resource for developing new products or their use as enrichment in various applications [52].

Li et al. [53] presented a study on the utilization of apple peel through fermentation using Aspergillus oryzae, resulting in the development of a new food ingredient in polyphenolic compounds, exhibiting significant antioxidant activity and prebiotic potential. Additionally, fungi have also been employed in the fermentations of grape peels, granadilla seeds, and apple pomace, which were fermented using *Penicillium* YZ-1, *Aspergillus niger* and *Zigomycetes fungi*, respectively [54,55,56]. These fungal fermentations have also been used to develop new food ingredients that can serve as food additives. Following the fermentation process, phenolic compounds with antioxidant properties can be extracted. These compounds may enrich foods with health-promoting bioactivities, including cholesterol adsorption capacity and pancreatic lipase inhibition, both of which are beneficial for addressing metabolic disorders in dietary interventions [57]. Ou et al. [58] opted for alcoholic fermentation using *Saccharomyces cerevisiae* var. FX10, followed by acetic fermentation with *Acetobacter malorum*, OQY-1, in the valorisation of lemon peel to develop new lemon-based vinegars, characterised by their aroma profile and an increase in volatile esters and alcohol compounds. Marula by-products (pomace and peel), derived from a southern African fruit, have also been utilised to develop valuable vinegars that exhibit high antioxidant capacity and are rich in phenolic compounds, demonstrating good acceptability [59].

In addition, the use of fruit peels in the enrichment and fortification of fermented foods has been one of the most frequently chosen options by researchers. Focusing on alcoholic fermentation, kiwifruit and orange peels have been valorised by using them in the fermentation of kiwifruit wine and beer, respectively [53,60]. The results were the development of new alcoholic-enriched beverages, highlighted by an increase in rose aroma in the volatile profile of kiwifruit wine, and an increase in total phenolics and antioxidants in enriched beers. By-products (bagasse) of taperebá, a tropical fruit, were also used to enrich sour beers, improving the aroma profile and resulting in a moderate alcoholic beverage [61]. Apple pomace has also been used to enrich cider, incorporated before alcoholic fermentation to enable the extraction of phenolic compounds. This resulted in a cider with a higher antioxidant capacity; it was lighter and had increased bitterness and a decrease in sourness [62]. The black grape pomace by-product was also used for the enrichment of shalgam juice, a Turkish non-alcoholic beverage fermented by yeast [63]. After the fermentation, black grape pomace enriched the beverage with tannins and phenolic compounds, increasing its health-promoting compounds and enhancing the final product. Other authors have used alcoholic fermentation to develop new enriched sourdoughs and breads using prickly pear peels [64]. The results include high titratable acidity and increased ethanol yield during the fermentation; this improved the quality of the baked products. Apple pomace by-product was also used in fermentation with *Fructilactobacillus florum* DSM 22689 and baker’s yeast to increase the nutritional value and organic acids of sourdough. Lactic acid fermentation is extensively used in the valorisation of fruit by-products, using a variety of LAB strains. For example, blueberry pomace was used to develop a novel lactofermented beverage using Lactobacillus casei as the starter. The fermented beverage was assessed through in vivo assays in mice, demonstrating enhancements in beneficial microbiota and short-chain fatty acids production, thereby strengthening the intestinal immune barrier [65]. Fermentation has also been developed for food fortification. In dairy products, grape, passion fruit, and pitaya by-products have been used to ferment whey to increase folate levels [66]. Qin et al. [67] used grape peels in the fermentation of milk with *Lacticaseibacillus paracasei* to produce fortified yogurt. The resulting product had increased thermal stability and improved rheological properties, including increased gel strength and hardness, along with elevated functional bioactivities, such as glucose-, cholesterol-, nitrite-adsorption capacities, and antioxidant capacities. Additionally, Vieira et al. [68] assessed the enrichment of fermented soy beverage using acerola by-products (seeds and peels) during the fermentation process. The bioactivity of this enriched beverage was evaluated through colonic fermentation using faecal samples from obese patients. Notable outcomes included modulation of the microbiota, with an increase in *Bifidobacterium* spp. and *Lactobacillus* spp., correlating with profiles observed in non-obese people [68].

In this context, both kombucha and kefir represent good examples of novel beverages with a market that continues to grow significantly. In 2019, the global market values for kombucha and kefir were estimated at USD 1.84 billion and USD 1.23 billion, respectively. These markets are projected to reach USD 10.45 billion for kombucha and USD 1.84 billion for kefir by 2027, with compound annual growth rates (CAGR) of 23.2% and 5.4%, respectively [69]. Microbiologically, both beverages are composed by a consortium of bacteria and yeasts. Kombucha is made from tea infusion and sugar and fermented by a symbiotic association of acetic acid bacteria (*Komagataeibacter*, *Acetobacter*, *Gluconacetobacter*, and *Gluconobacter* species) and yeasts (*Schizosaccharomyces*, *Candida*, *Zygosaccharomyces*, *Saccharomyces*, and *Brettanomyces*). In contrast, kefir is made from milk and fermented by LAB from the genera *Lactobacillus*, *Lactococcus*, *Leuconostoc*, and *Streptococcus* species, and yeast from the genera *Kluyveromyces* and *Saccharomyces* [69]. The development of this type of fermented fruit drinks or the use of fruit as a carrier for probiotics provides a compelling alternative for vegans, lactose-intolerant individuals, and those allergic to milk.

This rapidly growing market has also led to exploring the revalorisation of by-products, offering new opportunities for sustainable development whilst providing beverages with probiotic potential and high bioactive levels. For example, cocoa mucilage, coffee husk, and grape pomace by-products have been used in the development of kombucha [70,71,72,73]. Overall, the fermentation of these by-products results in kombucha-style beverages with high polyphenol and flavonoid content. This is mainly due to the extractability of polyphenols from the plant source, which increases the antioxidant capacity and specific functions such as increasing antibacterial and microbial safety [70], and anti-inflammatory and antidiabetic bioactivities [72]. Furthermore, the fermentation of these by-products contributes to the development of specific aro-ma profiles driven by the increase in volatile acids and ester compounds, which result in kombucha with good sensory acceptability [73]. De Menezes et al. [74] reported the revalorisation of acerola, strawberry and grape by-products (peels, seeds, and pomace) in the development of a novel water kefir-based beverage. The fermentation process, initiated by the inoculation of kefir grains, led to the identification of phytochemicals, including glucuronic, succinic, and glutaric acids, in the final product. They also observed enhanced antibacterial properties and antioxidant capacity, both of which may contribute to health benefits and extend the shelf life of the beverage. Other researchers [75] have worked with the combination of two by-products, utilising pineapple by-products (core) and whey protein to develop a novel fermented food through co-fermentation with LAB and yeast, resulting in increased dietary fibre and free amino acids, enhanced sensory qualities such as sourness and umami, and a richer volatile compound profile.

Alternatively, as previously mentioned, fruits and vegetables not meeting the aesthetic standards often do not reach the final consumer. These aesthetic rejections include defects such as vitreous texture, softness, growth scars, mechanical damage, bark defect, deformities, sun damage, smooth or discoloured skin, spots, and cracked surfaces. Several examples of the revalorisation of these “imperfect” fruits can be found in the scientific literature. For example, Salas-Millán et al. [46] used melons that exhibited defects such as deformities, cracks, scratches, or spots, or were not of a commercial size. The objective of that study was to develop a fruit-based wine from these substandard melons, focusing on their sugar content and flavour characteristics. The resulting melon wine retained the typical melon flavours and achieved good sensory acceptance. Furthermore, subsequent research demonstrated the scalability of this process for the production of a sparkling-style melon wine, thereby diversifying the revalorisation pathways for melon by-products [3].

In the same way, Saadoun et al. [76] reported the revalorisation of melons and oranges with aesthetic defects and their pomace to develop new food additives and flavours with higher floral, herbal green, and fruity notes for inclusion as additives in the development of food products and beverages. In the context of small-sized dis-carded kiwifruit, Hadj Saadoun et al. [77] identified effective starter cultures for optimising the production of methyl heptenone from kiwifruit biomass. Their fermentation conditions resulted in aromatically rich biomass that can be directly incorporated into food formulations or enriched with specific volatile compounds for use as flavouring agents. A further example involved the use of unripe and non-red tomatoes, discarded by farmers and retailers, with which Simoes et al. [78] reported the development of a new salad dressing, with good acceptability, based on a similar kefir fermentation using *L. plantarum*, *L. mesenteroides* and *K. marxianus*. The above examples highlight the potential for the revalorisation of fruit by-products through the use of fermentation for their conversion. Moreover, the revalorisation of fruit with aesthetic defects provides new sources of income for producers and offers economic diversification through circular economy strategies.

### 4.2. Vegetable By-Products

Vegetable and other leafy by-products have also been used in the development of new fermented beverages and other foods (Table 2). In particular, there are several examples of such by-products being used to produce new kombucha. For example, Marin-Gómez et al. [79] used leaves from different Brassica species such as *B. oleracea var*. *sabelica* × *B. oleracea var. gemmifera* and *Brassica oleracea var. capitata* and fermented them with SCOBY (Symbiotic Culture Of Bacteria and Yeast) inoculum. As a result, they obtained a kombucha-based beverage with high total phenolic compounds and antioxidant capacity. Another example that used Brassica species by-products in the development of kombucha was reported by Cánovas et al. [80], who reported the use of broccoli stalks fermented with commercial SCOBY inoculum to develop a kombucha enriched in glucosinolate and isothiocyanate compounds. On the other hand, Salas-Millán et al. [81] also used broccoli stalks in the development of new pickled vegetables. In that case, a spontaneous fermentation of the natural phytobiome of broccoli stalks was carried out which increased the content of health-promoting compounds such as phenolic acids, flavonoids and glucosinolates, with enhanced antioxidant capacity and global acceptability. Additionally, broccoli leaves were used in the development of a new lactofermented beverage by Salas-Millán et al. [9]. In that work, optimising the parameters of the fermentation process of L. plantarum enabled optimal levels of antioxidant capacity, phenolic compounds and microbial growth to be obtained. Thus, the resulting beverages were reported to have a high content of phenolic acids (chlorogenic acids), flavonoids (kaempferol derivatives) and glucosinolate hydrolysis (sulforaphane and indoles), and their anti-diabetic in vitro bioactivities were also reported due to its inhibition of α-amylase and α-glucosidase enzymes.

This section also includes other leafy by-products such as olive leaves [91] and pepper [95] in the development of a kombucha-style beverage and a vinegar, respectively. The fermentation of both by-products resulted in enriched kombucha with high levels of oleuropein and hydroxytyrosol, both of which are potent antioxidant compounds, and increased total phenolics in the vinegar. In addition, Song et al. [95] reported an increase in the inhibition of α-glucosidase, outlining the anti-diabetic potential of the vinegar made from leafy pepper discards. Kimoto-Nira et al. [94] fermented onion peels into a new valuable functional food with anti-hypertensive properties and which was rich in quercetin, providing an example of the valorisation of vegetables by-products into new products with health-promoting properties. As mentioned in Section 3, the selection of specific strains can enhance the profile of bioactive compounds. In this context, *Lactobacillus plantarum* and *Lactobacillus casei* significantly in-creased titratable acidity, lactic acid production, phenolic content, and carotenoid levels in carrot pomaces [93].

Wang et al. [96] used traditional spontaneous fermentation on asparagus by-products, specifically hard asparagus spears, reporting an increase in organic acids and free amino acids during fermentation, which enhanced flavour complexity, pro-moted a diverse and active microbial community, and improved the product’s preservation and health benefits. Other researchers have successfully extracted antimicrobial compounds from lactic acid fermentation of tomato, carrot, and melon by-products using various Lactobacillus strains, yielding extracts with significant antimicrobial activity against several pathogenic bacteria; this highlights a promising strategy for enhancing food preservation [87]. Similarly, lactic fermentation of artichoke by-products (bracts, stems, and leaves) enhanced the bioactivity of its extracts, providing a rich source of antimicrobial and antiviral compounds, such as cynarine, chlorogenic acid, caffeic acid, luteolin, and apigenin, which demonstrated efficacy against *Staphylococcus aureus*, *Bacillus cereus*, and the HSV-2 virus [92].

**Figure 3:**
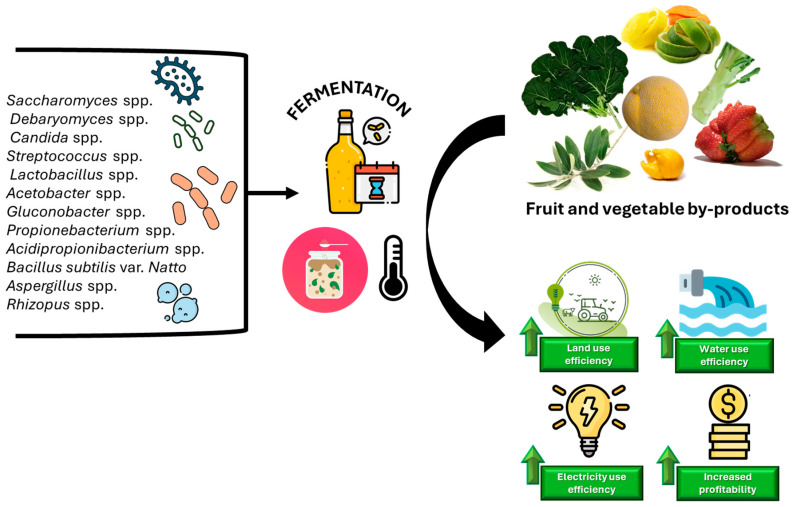
Transformation of fruit and vegetable by-products through fermentation with diverse microbial communities enhances resource efficiency—optimising electricity, land, and water use—and generates new market opportunities for companies by creating valuable fermented products.

Those previous studies provide examples of biomass revalorisation, highlighting types of plant biomass that are currently undervalued and left in the field. Revaluing this biomass can significantly improve resource efficiency by maximising the yield from inputs used in vegetable production, such as land, water and electricity. Reusing agro-industrial waste to create high-value-added products not only reduces the environmental impact but also presents an attractive opportunity for companies to explore new business lines (Figure 3) [97]. By contributing to waste reduction and improving the circular use of resources, these practices are essential for future applications and are driving commercial interest in sustainable food production.

## 5. Conclusions

This review highlights the potential of the fermentation process as an effective strategy for the revalorisation of fruit and vegetable by-products, offering a sustainable approach to reducing food loss and waste. Fermentation significantly contributes to environmental sustainability by minimising waste, optimising the use of resources, and providing an alternative to conventional waste treatment methods. The fermentation process enables the extraction of valuable phytochemical compounds that are present in these by-products, whilst modifying their flavour profiles. This results in the development of innovative food and beverage ingredients with appealing flavours and high consumer acceptance. Additionally, fermentation improves the nutritional value by enhancing bioactive compounds that benefit health. Furthermore, fermentation can extend the shelf life of foodstuffs, thereby making healthy food more accessible, particularly in developing countries where food storage is limited.

Fermentation can be utilised in the food sector in different ways, depending on the specific objectives and desired end products. Its applications include the preservation of foodstuffs for an extended shelf life, enhancing their flavour for consumer appeal, and fortifying their nutritional content to support health benefits. The approaches in question vary depending on the type of by-product (e.g., peels, seeds, pomace, and non-aesthetic unmarketable fruits and vegetables) and on the target product, which can range from beverages to enriched foods and novel food ingredients. However, research is currently somewhat limited as most developments in the scientific literature focus on laboratory-scale processes, which are often constrained by the seasonality of by-products and dependent on specific crops and processing times.

Future research should prioritise the development of scalable fermentation models and explore underutilised by-products with the objective of maximising the impact of fermentation. Addressing these research gaps will facilitate the implementation of fermentation in a broader range of real-world applications, with relevance for regions experiencing challenges in food accessibility. To further enhance consumer acceptance of fermented products, it will be essential to invest in consumer education and targeted marketing strategies, especially as many of these products may be unfamiliar to certain populations. Addressing these challenges can help the food sector realise the full potential of fermentation, with far-reaching implications for food security, environmental sustainability, and economic viability across the industry.

## Figures and Tables

**Table 2:** Revalorisation of fruit and vegetable by-products using fermentation as food processing.

Source	By-Product	Microorganism Involved	Developed Product	Outcomes	Reference
***Fruits***					
Grape, passion fruit, and pitaya	Pomace, peels, and seeds	*Lacticaseibacillus rhamnosus* LGG, *Bifidobacterium infantis* BB-02, and *Streptococcus thermophilus* TH-4	Fermented beverage with whey added	↑ Folate content	[66]
Tomate	Unripe and non-red tomatoes	*Lactobacillus plantarum*, *Leuconostoc mesenteroides*, *Kluyveromyces marxianus*	Salad dressing *	Good acceptability	[78]
Kiwifruit	Peel	*Saccharomyces cerevisiae*	Kiwifruit wine Enriched *	↑ Aroma Affects the clarification	[82]
Cocoa	Mucilage	Market yeast	Kombucha *	Good acceptability Total phenolic content	[71]
Black grape	Pomace	Yeast	Shalgam juice *	↑ Tannins ↑ Total polyphenolic content	[63]
Apple, blackcurrant	By-products	*Lacticaseibacillus casei*, *Liquorilactobacillus uvarum*	Marinade of poultry meat	↑ Microbiological safety ↓ pH ↓ Spermidine, phenylethylamine	[83]
Pineapple	Core	*Lactococcus lactis LA5*, *anseniaspora opuntiae SA2*	New fermented food *	↑ Dietary fibre ↑ Free amino acids ↓ Astringency and bitterness ↑ Umami, sourness, enriched ↑ Volatile compounds: floral, fruity, cheesy, among others	[75]
Apple	Apple peel	*Aspergillus oryzae*	Food ingredient *	↑ Polyphenolic content and antioxidant capacity ↑ Prebiotic potential	[53]
	Pomace	Alcoholic fermentation	Enriched cider *	↑ Total phenolic ↑ Antioxidant capacity ↑ Lightness and bitterness ↓ Sourness	[62]
	Pomace	*Zygomycetes fungi*	Food ingredient *	↑ γ-linolenic acid, carotenoid pigments, and phenolic antioxidants	[56]
		*Fructilactobacillus florum DSM 22689* Baker’s yeast	Sourdough	↑ Nutritional value ↑ Organic acids and fermentable sugars	[84]
Melon	Not reach cosmetical standards	*Saccharomyces cerevisiae*	Melon wine *	↑ Antioxidant capacity ↑ Aromatic profile Good acceptability	[3,46]
Kiwifruit	Small size	*Streptococcus porcorum 1980*, *Lacticaseibacillus paracasei 2243*, *Lactococcus lacti 2276*, *Companilactobacillus farciminis*, *Latilactobacillus curvatus 6180*, *Lactiplantibacillus plantarum 4193*, *Leuconostoc mesenteroides 6134*, *Hanseniaspora uvarum 6347*	Food ingredient, flavouring agent *	↑ Aroma profile	[77]
Orange	Peel	Yeast	Enriched beer *	↑ Colour ↑ Alcohol ↑ Total polyphenolic content, antioxidant capacity Good acceptability	[60]
Coffee	Coffee husk	*Brettanomyces bruxellensis*, *Saccharomyces cerevisiae*, *Komagataeibacter pomaceri*, and *Komagataeibacter rhaeticus*	Enriched kombucha *	↑ Total polyphenolic content, flavonoids, antioxidant capacity ↑ Antibacterial and microbial safety	[70]
		SCOBY		↑ Volatile acids and esters compounds Good acceptability	[73]
Grape	Peels	*Lacticaseibacillus paracasei*	Fortified yogurt	↑ Thermal stability ↑ Gel strength, hardness Stronger odour ↑ Functional bioactivities	[67]
		*Penicillium YZ-1*	Food ingredient *	↑ Viscosity, thermal stability ↑ Cholesterol adsorption capacity ↓ Lipase pancreatic	[54]
	Pomace	Kombucha consortia inoculum.	Kombucha *	↑ Anti-inflammatory activities ↑ Anti-diabetic activities ↑ Total phenolics and anthocyanins	[72]
Acerola, guava	By-products	Spontaneous, *L. casei* L-26, *L. fermentum* 56, *L. paracasei* 106, *L. plantarum* 53	Food ingredients *	↑ Total flavonoids, polyphenols, antioxidant	[85,86]
Lemon	Peel	*Saccharomyces cerevisiae var. FX10*, *Acetobacter malorum*, *OQY-1*	Vinegar *	↑ Volatile esters and alcohol compounds	[58]
Prickly pear fruit’	Peel	*Saccharomyces cerevisiae*	Sourdoughs, enriched bread	↑ Ethanol ↑ Total acidity	[64]
Taperebá or cará	Bagasse and juice fruit	*Saccharomyces cerevisiae*	Enriched sour beer *	Moderate alcohol content Positive aroma	[61]
Acerola	Peels, seeds, and pomace	Kefir grains	Kefir *	↑ Phytochemicals and compounds (glucuronic, succinic, and glutaric acids). ↑ Antibacterial potential ↑ Antioxidant activity	[74]
Strawberry					
Grape					
Melon and Orange	Aesthetic defects, pomace	*Lacticaseibacillus rhamnosus*	Aroma as food additives *	↑ Floral, herbal green, and fruity notes	[76]
Acerola	Seeds and peels	*L. acidophilus LA-5*, *B. longum BB-46*, *Streptococcus thermophilus TH4*	Enriched fermented soy beverage	↑ *Bifidobacterium* spp. and *Lactobacillus* spp. after colonic fermentation	[68]
Granadilla	Seeds	*Aspergillus niger*	Ingredient for food, cosmetic, and pharmaceutical industries *	↑ Total phenolic ↑ Total flavonoids ↑ Antioxidant capacity	[55]
Blueberry	Pomace	*Lactobacillus casei*	Fermented beverage *	↑ Beneficial microbiota in mice ↑ Short-chain fatty acids ↑ Intestinal immune barrier	[65]
Marula	Pomace and skin	Starter cultures and spontaneous	Vinegar *	↑ Total phenolics ↑ Antioxidant activity Good acceptability	[59]
Melon, tomato, and carrot	Pomace and discarded during harvest	*L. plantarum* (POM1 and 285), *L. casei* (2240 and 2246), *L. paracasei* (4186), and *L. rhamnosus* (1019 and 1473)	Food additive (antimicrobial compounds) *	↑ Antimicrobial activity: *Listeria monocytogenes*, *Salmonella* spp., *Escherichia coli*, *Staphylococcus Aureus*, and *Bacillus cereus*	[87]
Orange and mandarin	Peels	Lactoflora^®^, commercial culture	Enriched tarhana	↑ Oil absorption capacity, swelling capacity ↑ Dietary fibre Good acceptability for preparation if <10% of peels in flour	[88]
Passion fruit	Peel	*L. acidophilus* LAFTI L10, *L. casei* LAFTI L26, *B. animalis* subsp. *lactis* LAFTI B94, *L. fermentum OgiE1*, and *L. plantarum* A6	Enriched fermented soy milk	↓ Time of fermentation ↑ Amylase activity ↑ Exopolysaccharide	[89]
		*Streptococcus thermophilus*, *Lactobacillus delbrueckii* subsp. *bulgaricus* (CY340), *Lactobacillus acidophilus* (L10 and NCFM), *Bifidobacterium animalis* subsp. *lactis* (BI04 and HN019)	Enriched probiotic yoghurt	↓ Time of fermentation ↑ Total acidity ↑ Firmness, consistency, cohesiveness	[90]
***Vegetables and leafy***					
Brassica species *B. oleracea var. sabelica* × *B. oleracea var. Gemmifera* *Brassica oleracea var. capitata*	Leaves	SCOBY	Kombucha *	↑ Total phenolics ↑ Antioxidant capacity ↓ Lower sensorial acceptability than kombucha control	[79]
Broccoli	Stalks	Commercial SCOBY	Kombucha *	↑ Glucosinolates and isothiocyanate	[80]
	Stalk	Spontaneous	Pickled	↑ Glucosinolates ↑ Total polyphenols ↑ Flavonoids Good acceptability	[81]
	Leaves	*Lactiplantibacillus plantarum*	Lactofermented beverage *	↑ Total phenolics ↑ Isothiocyanates ↑ Indoles ↑ Antioxidant capacity ↑ Anti-diabetic potential	[9]
*Olea europaea* L	Leaves	Commercial SCOBY	Kombucha *	↑ Oleuropein and hydroxytyrosol	[91]
Artichoke	Leaves, stems, and outer bracts	*Lactobacillus casei* ATTC 3931; *L. plantarum* ATTC 8014; *L. casei subs. rhamnosus* ATCC 7469; *L. fermentum* ATCC 9338	Food additives (antimicrobial and antiviral constituents)	↑ Flavonoid content ↑ Antimicrobial and antiviral effect: *Staphylococcus aureus*, *Bacillus cereus*, and HSV-2 virus	[92]
Carrot	Pomace	*Lactobacillus acidophilus* LA-5, *Lactobacillus casei* 431, and *Lactobacillus plantarum* Harvest-LB1	Food ingredient *	↑ Phenolic acid content ↑ Anthocyanin content ↑ α-carotene	[93]
Onion	Peel	*Lactobacillus* strains (E19, E31, J11, J41, and 361)	Functional food	↑ Anti-hypertensive activity	[94]
Pepper	Leaves	*Lactobacillus homohiochii* JBCC 25 and JBCC46, *Saccharomyces cerevisiae* ATCC 18824, *Actobacter aceti* KACC 1978	Vinegar *	↑ Anti-diabetic potential ↑ Antioxidant activity ↑ Total phenolics	[95]

* The by-product used was discarded from the final fermented product. The symbols ↑/↓ indicate an increase or decrease, respectively, of the phytochemical or specified value. SCOBY: Symbiotic Culture Of Bacteria and Yeast.

## Data Availability

No new data were created or analyzed in this study. Data sharing is not applicable to this article.

## References

[1] FAO (2019). The State of Food and Agriculture: Moving Forward on Food Loss and Waste Reduction.

[2] Gustavsson J., Cederberg C., Sonesson U. (2011). Global Food Loss and Food Waste.

[3] Salas-Millán J.Á., Aguayo E., Conesa-Bueno A., Aznar A. (2023). Revalorization of Melon By-Product to Obtain a Novel Sparkling Fruity-Based Wine. Foods.

[4] FAO (2023). The State of Food Security and Nutrition in the World 2023.

[5] Wunder S., McFarland K., Hirschnitz-Garbers M., Parfitt J.L.K., Jarosz D., Youhanan L., Stenmarck A., Colin F., Burgos S., Gheoldus M. (2018). Food Waste Prevention and Valorisation: Relevant EU Policy Areas.

[6] The Courtauld Commitment 2030|WRAP. https://www.wrap.ngo/taking-action/food-drink/initiatives/courtauld-commitment.

[7] EU Platform on Food Losses and Food Waste (2019). Redistribution of Surplus Food: Examples of Practices in the Member States.

[8] El Gobierno Pone En Marcha El Proyecto de Ley de Prevención de Las Pérdidas y El Desperdicio Alimentario. https://www.mapa.gob.es/es/prensa/ultimas-noticias/el-gobierno-pone-en-marcha-el-proyecto-de-ley-de-prevenci%C3%B3n-de-las-p%C3%A9rdidas-y-el-desperdicio-alimentario/tcm:30-673097.

[9] Salas-Millán J.Á., Conesa-Bueno A., Aguayo E. (2024). A Novel Antidiabetic Lactofermented Beverage from Agro-Industrial Waste (Broccoli Leaves): Process Optimisation, Phytochemical Characterisation, and Shelf-Life through Thermal Treatment and High Hydrostatic Pressure. Food Biosci..

[10] De Ancos B., Colina-Coca C., González-Peña D., Sánchez-Moreno C., Gupta V.K., Tuohy M.G. (2015). Bioactive Compounds from Vegetable and Fruit By-Products. Biotechnology of Bioactive Compounds: Sources and Applications.

[11] Tamang J.P., Cotter P.D., Endo A., Han N.S., Kort R., Liu S.Q., Mayo B., Westerik N., Hutkins R. (2020). Fermented Foods in a Global Age: East Meets West. Compr. Rev. Food Sci. Food Saf..

[12] Marco M.L., Heeney D., Binda S., Cifelli C.J., Cotter P.D., Foligné B., Gänzle M., Kort R., Pasin G., Pihlanto A. (2017). Health Benefits of Fermented Foods: Microbiota and Beyond. Curr. Opin. Biotechnol..

[13] Bamforth C.W., Cook D.J. (2019). Food, Fermentation, and Micro-Organisms.

[14] Voidarou C., Antoniadou M., Rozos G., Tzora A., Skoufos I., Varzakas T., Lagiou A., Bezirtzoglou E. (2020). Fermentative Foods: Microbiology, Biochemistry, Potential Human Health Benefits and Public Health Issues. Foods.

[15] Cano-Lamadrid M., Artés-Hernández F. (2021). By-Products Revalorization with Non-Thermal Treatments to Enhance Phytochemical Compounds of Fruit and Vegetables Derived Products: A Review. Foods.

[16] Aidoo K.E., Robert Nout M.J. (2010). Functional Yeasts and Molds in Fermented Foods and Beverages. Fermented Foods and Beverages of the World.

[17] Duan S.F., Han P.J., Wang Q.M., Liu W.Q., Shi J.Y., Li K., Zhang X.L., Bai F.Y. (2018). The Origin and Adaptive Evolution of Domesticated Populations of Yeast from Far East Asia. Nat. Commun..

[18] Stewart G.G., Batt C.A., Tortorello M.L. (2014). SACCHAROMYCES|Saccharomyces cerevisiae. Encyclopedia of Food Microbiology.

[19] Albergaria H., Arneborg N. (2016). Dominance of *Saccharomyces cerevisiae* in Alcoholic Fermentation Processes: Role of Physiological Fitness and Microbial Interactions. Appl. Microbiol. Biotechnol..

[20] de Celis M., Ruiz J., Martín-Santamaría M., Alonso A., Marquina D., Navascués E., Gómez-Flechoso M., Belda I., Santos A. (2019). Diversity of Saccharomyces Cerevisiae Yeasts Associated to Spontaneous and Inoculated Fermenting Grapes from Spanish Vineyards. Lett. Appl. Microbiol..

[21] Eder M., Sanchez I., Brice C., Camarasa C., Legras J.L., Dequin S. (2018). QTL Mapping of Volatile Compound Production in Saccharomyces Cerevisiae during Alcoholic Fermentation. BMC Genom..

[22] Ruiz J., Kiene F., Belda I., Fracassetti D., Marquina D., Navascués E., Calderón F., Benito A., Rauhut D., Santos A. (2019). Effects on Varietal Aromas during Wine Making: A Review of the Impact of Varietal Aromas on the Flavor of Wine. Appl. Microbiol. Biotechnol..

[23] Noble J.J., Sanchez I.I., Blondin B.B. (2015). Identification of New Saccharomyces Cerevisiae Variants of the MET2 and SKP2 Genes Controlling the Sulfur Assimilation Pathway and the Production of Undesirable Sulfur Compounds during Alcoholic Fermentation. Microb. Cell Fact..

[24] Ehrlich F. (1907). Über Die Bedingungen Der Fuselölbildung Und Über Ihren Zusammenhang Mit Dem Eiweißaufbau Der Hefe. Berichte Der Dtsch. Chem. Ges..

[25] Diez-Simon C., Ammerlaan B., van den Berg M., van Duynhoven J., Jacobs D., Mumm R., Hall R.D. (2020). Comparison of Volatile Trapping Techniques for the Comprehensive Analysis of Food Flavourings by Gas Chromatography-Mass Spectrometry. J. Chromatogr. A.

[26] Dias D.R., Duarte W.F., Schwan R.F. (2017). Methods of Evaluation of Fruit Wines. Science and Technology of Fruit Wine Production.

[27] Fabbri L.P., Cavallero A., Vidotto F., Gabriele M. (2024). Bioactive Peptides from Fermented Foods: Production Approaches, Sources, and Potential Health Benefits. Foods.

[28] Bruner J., Fox G. (2020). Novel Non-Cerevisiae Saccharomyces Yeast Species Used in Beer and Alcoholic Beverage Fermentations. Fermentation.

[29] Park M.K., Seo J.A., Kim Y.S. (2019). Comparative Study on Metabolic Changes of Aspergillus Oryzae Isolated from Fermented Foods According to Culture Conditions. Int. J. Food Microbiol..

[30] Venturini Copetti M. (2019). Yeasts and Molds in Fermented Food Production: An Ancient Bioprocess. Curr. Opin. Food Sci..

[31] LPSN—List of Prokaryotic Names with Standing in Nomenclature. https://lpsn.dsmz.de/.

[32] Zheng J., Wittouck S., Salvetti E., Franz C.M.A.P., Harris H.M.B., Mattarelli P., O’toole P.W., Pot B., Vandamme P., Walter J. (2020). A Taxonomic Note on the Genus Lactobacillus: Description of 23 Novel Genera, Emended Description of the Genus Lactobacillus Beijerinck 1901, and Union of Lactobacillaceae and Leuconostocaceae. Int. J. Syst. Evol. Microbiol..

[33] Daba G.M., Elkhateeb W.A. (2020). Bacteriocins of Lactic Acid Bacteria as Biotechnological Tools in Food and Pharmaceuticals: Current Applications and Future Prospects. Biocatal. Agric. Biotechnol..

[34] Koutsoumanis K., Allende A., Alvarez-Ordonez A., Bolton D., Bover-Cid S., Chemaly M., De Cesare A., Hilbert F., Lindqvist R., EFSA BIOHAZ Panel (2024). EFSA Updated List of QPS-Recommended Microorganisms for Safety Risk Assessments Carried Out by EFSA.

[35] Raj T., Chandrasekhar K., Kumar A.N., Kim S.H. (2021). Recent Biotechnological Trends in Lactic Acid Bacterial Fermentation for Food Processing Industries. Syst. Microbiol. Biomanuf..

[36] Wu W., Li H. (2018). Metabolites of Lactic Acid Bacteria. Lactic Acid Bacteria in Foodborne Hazards Reduction: Physiology to Practice.

[37] Barbosa J., Borges S., Teixeira P. (2014). Selection of Potential Probiotic Enterococcus Faecium Isolated from Portuguese Fermented Food. Int. J. Food Microbiol..

[38] Özmen Toǧay S., Çelebi Keskin A., Açik L., Temiz A. (2010). Virulence Genes, Antibiotic Resistance and Plasmid Profiles of *Enterococcus faecalis* and *Enterococcus faecium* from Naturally Fermented Turkish Foods. J. Appl. Microbiol..

[39] Thierry A., Deutsch S.M., Falentin H., Dalmasso M., Cousin F.J., Jan G. (2011). New Insights into Physiology and Metabolism of *Propionibacterium freudenreichii*. Int. J. Food Microbiol..

[40] Torino M.I., Taranto M.P., Font De Valdez G. (2005). Citrate Catabolism and Production of Acetate and Succinate by *Lactobacillus helveticus* ATCC 15807. Appl. Microbiol. Biotechnol..

[41] Aubourg S.P., Caballero B., Finglas P.M., Toldrá F. (2016). Fish: Processing. Encyclopedia of Food and Health.

[42] Zapaśnik A., Sokołowska B., Bryła M. (2022). Role of Lactic Acid Bacteria in Food Preservation and Safety. Foods.

[43] Stanojević-Nikolić S., Dimić G., Mojović L., Pejin J., Djukić-Vuković A., Kocić-Tanackov S. (2016). Antimicrobial Activity of Lactic Acid Against Pathogen and Spoilage Microorganisms. J. Food Process Preserv..

[44] Vignolo G., Saavedra L., Sesma F., Raya R., Bhat R., Alias A.K., Paliyath G. (2012). Food Bioprotection: Lactic Acid Bacteria as Natural Preservatives. Progress in Food Preservation.

[45] Ruiz Rodríguez L.G., Zamora Gasga V.M., Pescuma M., Van Nieuwenhove C., Mozzi F., Sánchez Burgos J.A. (2021). Fruits and Fruit By-Products as Sources of Bioactive Compounds. Benefits and Trends of Lactic Acid Fermentation in the Development of Novel Fruit-Based Functional Beverages. Food Res. Int..

[46] Salas-Millán J.Á., Aznar A., Conesa E., Conesa-Bueno A., Aguayo E. (2022). Fruit Wine Obtained from Melon By-Products: Physico-Chemical and Sensory Analysis, and Characterization of Key Aromas by GC-MS. Foods.

[47] Bajzelj B., McManus W. Andrew Parry Food Waste in Primary Production in the UK|WRAP. https://www.wrap.ngo/resources/report/food-waste-primary-production-uk.

[48] Tarazona-Díaz M.P., Aguayo E. (2013). Assessment of By-Products from Fresh-Cut Products for Reuse as Bioactive Compounds. Food Sci. Technol. Int..

[49] Laufenberg G., Kunz B., Nystroem M. (2003). Transformation of Vegetable Waste into Value Added Products:: (A) the Upgrading Concept; (B) Practical Implementations. Bioresour. Technol..

[50] Tarazona-Díaz M.P., Viegas J., Moldao-Martins M., Aguayo E. (2011). Bioactive Compounds from Flesh and By-Product of Fresh-Cut Watermelon Cultivars. J. Sci. Food Agric..

[51] Ayala-Zavala J.F., Vega-Vega V., Rosas-Domínguez C., Palafox-Carlos H., Villa-Rodriguez J.A., Siddiqui M.W., Dávila-Aviña J.E., González-Aguilar G.A. (2011). Agro-Industrial Potential of Exotic Fruit Byproducts as a Source of Food Additives. Food Res. Int..

[52] Hussain H., Mamadalieva N.Z., Hussain A., Hassan U., Rabnawaz A., Ahmed I., Green I.R. (2022). Fruit Peels: Food Waste as a Valuable Source of Bioactive Natural Products for Drug Discovery. Curr. Issues Mol. Biol..

[53] Li J., Ye F., Zhou Y., Lei L., Chen J., Li S., Zhao G. (2024). Tailoring the Composition, Antioxidant Activity, and Prebiotic Potential of Apple Peel by Aspergillus Oryzae Fermentation. Food Chem. X.

[54] Ma W., Liang Y., Lin H., Chen Y., Xie J., Ai F., Yan Z., Hu X., Yu Q. (2023). Fermentation of Grapefruit Peel by an Efficient Cellulose-Degrading Strain, (Penicillium YZ-1): Modification, Structure and Functional Properties of Soluble Dietary Fiber. Food Chem..

[55] Santos T.R.J., Feitosa P.R.B., Gualberto N.C., Narain N., Santana L.C.L.A. (2021). Improvement of Bioactive Compounds Content in Granadilla (*Passiflora ligularis*) Seeds after Solid-State Fermentation. Food Sci. Technol. Int..

[56] Dulf F.V., Vodnar D.C., Dulf E.H. (2023). Solid-State Fermentation with Zygomycetes Fungi as a Tool for Biofortification of Apple Pomace with γ-Linolenic Acid, Carotenoid Pigments and Phenolic Antioxidants. Food Res. Int..

[57] Trisat K., Wong-on M., Lapphanichayakool P., Tiyaboonchai W., Limpeanchob N. (2017). Vegetable Juices and Fibers Reduce Lipid Digestion or Absorption by Inhibiting Pancreatic Lipase, Cholesterol Solubility and Bile Acid Binding. Int. J. Veg. Sci..

[58] Ou Q., Zhao J., Sun Y., Zhao Y., Zhang B. (2023). Utilization of Lemon Peel for the Production of Vinegar by a Combination of Alcoholic and Acetic Fermentations. Foods.

[59] Molelekoa T.B.J., Regnier T., Da Silva L.S., Augustyn W.A. (2018). Potential of Marula (*Sclerocarya birrea* Subsp. *caffra*) Waste for the Production of Vinegar through Surface and Submerged Fermentation. S. Afr. J. Sci..

[60] Dobón-Suárez A., Giménez M.J., Gutiérrez-Pozo M., Zapata P.J. (2024). Development of New Craft Beer Enriched with a Byproduct of Orange. Acta Hortic..

[61] Praia A.B., Herkenhoff M.E., Broedel O., Frohme M., Saad S.M.I. (2022). Sour Beer with *Lacticaseibacillus paracasei* Subsp. *paracasei* F19: Feasibility and Influence of Supplementation with *Spondias mombin* L. Juice and/or By-Product. Foods.

[62] Bortolini D.G., Benvenutti L., Demiate I.M., Nogueira A., Alberti A., Zielinski A.A.F. (2020). A New Approach to the Use of Apple Pomace in Cider Making for the Recovery of Phenolic Compounds. LWT.

[63] Akbulut M., Çoklar H., Bulut A.N., Hosseini S.R. (2024). Evaluation of Black Grape Pomace, a Fruit Juice by-Product, in Shalgam Juice Production: Effect on Phenolic Compounds, Anthocyanins, Resveratrol, Tannin, and in Vitro Antioxidant Activity. Food Sci. Nutr..

[64] Chafai Y., Raffak A., El-Aalaoui M., Sbaghi M., Djerrari A., Zahar M. (2023). Valorization of Prickly Pear Peels & Seed Press-Cake in Traditional Sourdoughs and Evaluation of Their Bread-Making Capacities. J. Microbiol. Biotechnol. Food Sci..

[65] Cheng Y., Tang S., Huang Y., Liang F., Fang Y., Pan S., Wu T., Xu X. (2020). Lactobacillus Casei-Fermented Blueberry Pomace Augments SIgA Production in High-Fat Diet Mice by Improving Intestinal Microbiota. Food Funct..

[66] Cucick A.C.C., Obermaier L., Galvão E.F., Suzuki J.Y., Nascimento K.R., Fabi J.P., Rychlik M., de Melo Franco B.D.G., Saad S.M.I. (2024). Integrating Fruit By-Products and Whey for the Design of Folate-Bioenriched Innovative Fermented Beverages Safe for Human Consumption. Int. J. Food Microbiol..

[67] Qin X., Yang C., Si J., Chen Y., Xie J., Tang J., Dong X., Cheng Y., Hu X., Yu Q. (2023). Fortified Yogurt with High-Quality Dietary Fiber Prepared from the by-Products of Grapefruit by Superfine Grinding Combined with Fermentation Treatment. LWT.

[68] Vieira A.D.S., de Souza C.B., Padilha M., Zoetendal E.G., Smidt H., Saad S.M.I., Venema K. (2021). Impact of a Fermented Soy Beverage Supplemented with Acerola By-Product on the Gut Microbiota from Lean and Obese Subjects Using an in Vitro Model of the Human Colon. Appl. Microbiol. Biotechnol..

[69] Chong A.Q., Lau S.W., Chin N.L., Talib R.A., Basha R.K. (2023). Fermented Beverage Benefits: A Comprehensive Review and Comparison of Kombucha and Kefir Microbiome. Microorganisms.

[70] Nguyen Le B.X., Van T.P., Phan Q.K., Pham G.B., Quang H.P., Do A.D. (2024). Coffee Husk By-Product as Novel Ingredients for Cascara Kombucha Production. J. Microbiol. Biotechnol..

[71] Rodríguez-Castro R., Guerrero R., Valero A., Franco-Rodriguez J., Posada-Izquierdo G. (2024). Cocoa Mucilage as a Novel Ingredient in Innovative Kombucha Fermentation. Foods.

[72] Barakat N., Bouajila J., Beaufort S., Rizk Z., Taillandier P., Bartkiene E., Barakat N., Bouajila J., Beaufort S., Rizk Z. (2024). Development of a New Kombucha from Grape Pomace: The Impact of Fermentation Conditions on Composition and Biological Activities. Beverages.

[73] Sales A.L., Cunha S.C., Morgado J., Cruz A., Santos T.F., Ferreira I.M.P.L.V.O., Fernandes J.O., Miguel M.A.L., Farah A. (2023). Volatile, Microbial, and Sensory Profiles and Consumer Acceptance of Coffee Cascara Kombuchas. Foods.

[74] de Menezes J.L., Mizuta A.G., Dutra T.V., Ferreira T.V., Bonin E., Castro J.C., Schipfer C.W.T., Szczerepa M.M.D.A., Lancheros C.A.C., Pilau E.J. (2022). Kefir Fermented Fruit By-Products: Anti-*Alicyclobacillus* spp. Activity, and Antioxidant Activity. Food Sci. Technol..

[75] Luo J.W., Xiao S., Suo H., Wang B., Cai Y.X., Wang J.H. (2024). Dynamics of Nutrients, Sensory Quality and Microbial Communities and Their Interactions during Co-Fermentation of Pineapple by-Products and Whey Protein. Food Chem. X.

[76] Hadj Saadoun J., Ricci A., Cirlini M., Bancalari E., Bernini V., Galaverna G., Neviani E., Lazzi C. (2021). Production and Recovery of Volatile Compounds from Fermented Fruit By-Products with *Lacticaseibacillus rhamnosus*. Food Bioprod. Process..

[77] Hadj Saadoun J., Del Vecchio L., Bettera L., Fontechiari L., Martelli F., Ricci A., Levante A., Bancalari E., Cirlini M., Lazzi C. (2024). Design of Experiment Approach to Boost Volatile Production from Kiwi Byproducts. Bioresour. Technol..

[78] Simões S., Santos R., Sousa I., Prista C., Raymundo A. (2024). Fermented Unripe Tomato Paste—Development of Innovative Salad Dressings as a Contribution to Circular Economy. Food Sci. Technol. Int..

[79] Martin-Gómez H., Diez M., Abadias M., Rivera A., Aguiló-Aguayo I. (2024). Promoting a Circular Economy by Developing New Gastronomic Products from Brassica Non-Edible Leaves. Int. J. Food Sci. Technol..

[80] Cánovas B.M., García-Viguera C., Medina S., Domínguez-Perles R. (2023). ‘Kombucha’-like Beverage of Broccoli By-Products: A New Dietary Source of Bioactive Sulforaphane. Beverages.

[81] Salas-Millán J.Á., Aznar A., Conesa E., Conesa-Bueno A., Aguayo E. (2022). Functional Food Obtained from Fermentation of Broccoli By-Products (Stalk): Metagenomics Profile and Glucosinolate and Phenolic Compounds Characterization by LC-ESI-QqQ-MS/MS. LWT.

[82] Zhou B., Liu X., Lan Q., Wan F., Yang Z., Nie X., Cai Z., Hu B., Tang J., Zhu C. (2024). Comparison of Aroma and Taste Profiles of Kiwi Wine Fermented with/without Peel by Combining Intelligent Sensory, Gas Chromatography-Mass Spectrometry, and Proton Nuclear Magnetic Resonance. Foods.

[83] Klementaviciute J., Zavistanaviciute P., Klupsaite D., Rocha J.M., Gruzauskas R., Viskelis P., El Aouad N., Bartkiene E. (2024). Valorization of Dairy and Fruit/Berry Industry By-Products to Sustainable Marinades for Broilers’ Wooden Breast Meat Quality Improvement. Foods.

[84] Martău G.A., Teleky B.E., Ranga F., Pop I.D., Vodnar D.C. (2021). Apple Pomace as a Sustainable Substrate in Sourdough Fermentation. Front. Microbiol..

[85] de Oliveira S.D., de Souza E.L., Araújo C.M., Martins A.C.S., da Silva Campelo Borges G., dos Santos Lima M., Viera V.B., Garcia E.F., da Conceição M.L., de Souza A.L. (2023). Spontaneous Fermentation Improves the Physicochemical Characteristics, Bioactive Compounds, and Antioxidant Activity of Acerola (*Malpighia emarginata* D.C.) and Guava (*Psidium guajava* L.) Fruit Processing by-Products. 3 Biotech.

[86] de Oliveira S.D., Araújo C.M., da Silva Campelo Borges G., dos Santos Lima M., Viera V.B., Garcia E.F., de Souza E.L., de Oliveira M.E.G. (2020). Improvement in Physicochemical Characteristics, Bioactive Compounds and Antioxidant Activity of Acerola (*Malpighia emarginata* D.C.) and Guava (*Psidium guajava* L.) Fruit by-Products Fermented with Potentially Probiotic Lactobacilli. LWT.

[87] Ricci A., Bernini V., Maoloni A., Cirlini M., Galaverna G., Neviani E., Lazzi C. (2019). Vegetable By-Product Lacto-Fermentation as a New Source of Antimicrobial Compounds. Microorganisms.

[88] Magala M., Kohajdová Z., Karovičová J., Šubová A. (2015). Utilization of Citrus Crops Processing By-Products in the Preparation of Tarhana. Potravin. Slovak J. Food Sci..

[89] do Espirito-Santo A.P., Mouquet-Rivier C., Humblot C., Cazevieille C., Icard-Vernière C., Soccol C.R., Guyot J.P. (2014). Influence of Cofermentation by Amylolytic Lactobacillus Strains and Probiotic Bacteria on the Fermentation Process, Viscosity and Microstructure of Gruels Made of Rice, Soy Milk and Passion Fruit Fiber. Food Res. Int..

[90] do Espírito Santo A.P., Perego P., Converti A., Oliveira M.N. (2012). Influence of Milk Type and Addition of Passion Fruit Peel Powder on Fermentation Kinetics, Texture Profile and Bacterial Viability in Probiotic Yoghurts. LWT.

[91] Lazzaroli C., Sordini B., Daidone L., Veneziani G., Esposto S., Urbani S., Selvaggini R., Servili M., Taticchi A. (2023). Recovery and Valorization of Food Industry By-Products through the Application of *Olea europaea* L. Leaves in Kombucha Tea Manufacturing. Food Biosci..

[92] Cioni E., Di Stasi M., Iacono E., Lai M., Quaranta P., Luminare A.G., Gambineri F., De Leo M., Pistello M., Braca A. (2024). Enhancing Antimicrobial and Antiviral Properties of *Cynara scolymus* L. Waste through Enzymatic Pretreatment and Lactic Fermentation. Food Biosci..

[93] Uzun D.E., Dikmetas D.N., Karbancioglu-Guler F., Tomas M., Capanoglu E. (2024). Exploring the Impact of Fermentation on Bioactive Compounds in Two Different Types of Carrot Pomace. Food Biosci..

[94] Kimoto-Nira H., Ohashi Y., Amamiya M., Moriya N., Ohmori H., Sekiyama Y. (2020). Fermentation of Onion (*Allium cepa* L.) Peel by Lactic Acid Bacteria for Production of Functional Food. J. Food Meas. Charact..

[95] Song Y.R., Shin N.S., Baik S.H. (2014). Physicochemical Properties, Antioxidant Activity and Inhibition of α-Glucosidase of a Novel Fermented Pepper (*Capsiccum annuum* L.) Leaves-Based Vinegar. Int. J. Food Sci. Technol..

[96] Wang L., Huang J., Hu S., Li X., Zhang Y., Cheng W., Yuan L., Liu G. (2025). The Dynamic Changes and Correlations between Biochemical Properties, Flavor and Microbial Community during Fermentation of Asparagus by-Products. Food Chem..

[97] Fernández-Ochoa Á., Leyva-Jiménez F.J., Pimentel-Moral S., del Carmen Villegas-Aguilar M., Alañón M.E., Segura-Carretero A., de la Luz Cádiz-Gurrea M. (2021). Revalorisation of Agro-Industrial Wastes into High Value-Added Products. Advances in Science, Technology and Innovation.

